# Turning data into insights in Jub, an extensible generic big data platform for life science and healthcare applications

**DOI:** 10.1038/s41598-025-32196-3

**Published:** 2025-12-19

**Authors:** Ignacio Castillo-Barrios, Melesio Crespo-Sanchez, Hugo G. Reyes-Anastacio, Jose L. Gonzalez-Compean, Ivan Lopez-Arevalo, J. Armando Barron-Lugo, J. Carlos Morin-Garcia, Yelda A. Leal, Jaqueline Calderon-Hernandez, Heriberto Aguirre-Meneses, Marco Antonio Núñez-Gaona

**Affiliations:** 1 Cinvestav Tamaulipas, Victoria, 87130 Tamaulipas México; 2 Coordination for Innovation and Application of Science and Technology (CIACYT), Autonomous University of San Luis Potosí, Avenida Sierra Leona No. 550, Colonia Lomas Segunda Sección, San Luis Potosí, 78210 Mexico; 3 Centro Institucional de Capacitación y Registro de Cáncer (CICyRC), UMAE-Merida, Instituto Mexicano del Seguro Social (IMSS), Merida, Yucatan Mexico; 4 Coordinación de Registros de Cáncer de Base Poblacional, Coordinación de Investigación en Salud (CIS), Instituto Mexicano de Seguro Social (IMSS), Ciudad de Mexico, Mexico; 5 Instituto Nacional de Rehabilitación Luis Guillermo Ibarra Ibarra, Departamento de Sistemas Médicos, Ciudad de México, 14389 México; 6 Dpto. de Computación e Ingeniería Industrial, Universidad de Monterrey, Ave. Ignacio Morones Prieto, 66238 San Pedro Garza García, Nuevo León México

**Keywords:** Life Science and Healthcare Data Platform, Decision-making processes, Data observatories, FAIR software, Cancer, Environmental sciences, Environmental social sciences, Diseases, Health care, Risk factors, Mathematics and computing

## Abstract

This paper presents *Jub*, a Life Science and Healthcare Data Platform (LSHDP) based on generic sandboxes that integrate AI tools and cloud storage into big data science services. *Jub* automatically and transparently creates data science services to transform datasets into massive information products by using a profiling methodology. These products are presented by generic-secure cloud-based FAIR observatories adding Programmable, Configurable/Customizing, Adaptable, and Resiliency properties (PCA-FAIR-R). This enables organizations to conduct and customize complex analytics processes to support decision-making. We conducted a study case to convert mortality, climate, and pollutants datasets (2000-2023) reported by the Mexican Government into a solid core hub of information products: 16 strategic data observatories based on 85,171,404 information products created from 114,155,622 spatio-temporal profiles of the *International Classification of Diseases* (ICD-10) mortality classes/strata and cancerogenic substances. An exploratory study revealed highlights about the significance of breast cancer mortality rate growth showing possible associations with air pollutants. This paper also describes the lessons learned from the practice and experience of implementing *Jub* sandboxes-based observatories for the *Population-based Cancer Registry Network* deployed on the Mexican territory in 12 Mexican states by public healthcare institutions, as well as to implement bone cancer deep-learning-based diagnosis at a national Hospital.

## Introduction

The preparation, analysis, and management of a data cumulus –an aggregation of diverse datasets from multiple sources– are essential for supporting Data-Driven Decision-Making (*DDDM*) processes through valuable information products (e.g., plots, maps, and tables)^1,2^. Traditional *DDDM* involves three key tasks: *a)* acquiring data^3^; *b)* transforming data into reliable information products^4^; and *c)* disseminating and visualizing information to provide decision-makers with actionable insights^5,6^. These tasks are further complicated when complex *DDDM* scenarios require multiple data views across domains like healthcare, earth observation, and risk analysis^7–9^. Such platforms must also ensure data security and availability through advanced cloud storage and security services^10^. Life Science and Healthcare Data Platforms^11,12^ (*LSHDP*s) are emerging as solutions to help organizations create heterogeneous data repositories for generating high-value information products and integrated data views that reveal patterns and correlations across different datasets^2,11,13^. Configuring a *LSHDP* is challenging and often delegated to cloud service providers, leading to issues like *vendor lock-in* and heavy reliance on service staff^14–18^. In sensitive domains like healthcare, retaining control over data and processes throughout the data analysis lifecycle is essential.

This paper focuses on the development of *Jub*, a flexible *LSHDP* that enables organizations to create *analytics sandboxes* –a software component, the technological base of an observatory in a specific domain (mortality, pollutants, illnesses, etc.)– for analyzing large datasets and generating high-level information products supporting the creation of secure, interoperable cloud-based observatories, which facilitates collaborative data sharing across domains. At an executive level, a *sandbox* is the foundation for implementing an observatory that consolidates and curates information products specific to a particular domain. Based on *Jub*, observatories were implemented for the *Population-based Cancer Registry Network* (PCNR, see Appendix 7) to explore spatio-temporal mortality patterns and pollutant correlations. Additionally, *Jub* is an open-source platform released under the MIT license. All components —including source code, deployment scripts, and documentation— are available upon request to support reproducibility, extensibility, and reuse by the scientific community; this also enables transparent tracking of modifications to the platform and effective version control of this software. *Jub* is not intended as a commercial product, but rather as a research-driven, community-oriented platform to support reproducible, FAIR-aligned data analysis pipelines. Its design was shaped through close collaboration with public health institutions in Mexico, focusing on open science, transparency, and accessibility.

The contributions of this work are summarized as follows:

1. A platform model that allows the creation and management of observatories designed according to *FAIR* (Findable, Accessible, Interoperable, Reusable) principles^19,20^, with additional *PCA* and *R* features to enhance practical data processing (*PCA-FAIR-R*):
  *Programmable* (P): Observatories are managed automatically through code, eliminating the need for cloud provider intervention.
  *Configurable* (C): High-level code defines observatory variables and allows dataset modification, fusion, and merging.
  *Adaptable* (A): Changes to datasets, interfaces, or methods are dynamic and online, unlike static traditional observatories.
  *Resilient* (R): Security protocols and policies ensure data control, reduce *vendor lock-in*, and enhance fault tolerance, secure sharing, and reproducibility.
2. The proposed platform has been implemented as a comprehensive data science service, supporting 16 strategic data observatories. It leverages a vast repository of 85,171,404 information products derived from 114,155,622 spatio-temporal profiles, encompassing mortality categories and strata defined by the International Classification of Diseases (ICD-10) and data on carcinogenic substances. Each profile represents a collection of records sharing common variable combinations. For example, a possible profile would be: *women over the age of 20 residing in Mexico City who succumbed to breast cancer in 2022*, whereas another profile is *the amount of emissions of Arsenic reported in Mexico City for 2022*. In this case, both profiles are events arising in the same space and temporal values.
3. A case study examining the relationship between breast cancer mortality and air pollutants in Mexico. This case study highlights the platform’s capability to integrate, process, and analyze heterogeneous datasets.

The paper is structured as follows: Section Related work discusses related work; Section Conceptual architecture presents *Jub*’s conceptual architecture; Section Case study: cancer mortality and air pollutants in Mexico details a case study; Section Learned lessons outlines learned lessons; and Section Conclusions provides conclusions of this work.

## Related work

Current solutions in data management focus on different aspects such as curated data management, massive data processing, or data visualization^21,22^. However, these solutions are typically developed for specific purposes^5^, with limited adaptability to other domains or data types (e.g., signals or imagery). Additionally, data fusion remains constrained by ad hoc development processes, often leading organizations to build separate data observatories or analytic processes for each case, which is not feasible for strategic information analysis.

This section presents a qualitative study comparing state-of-the-art approaches to *Jub*, focusing on key properties for building, managing, deploying, and using observatories based on retrieval systems for information products. The comparison considers three categories:

1. *Dynamic Properties*: Measure the flexibility in automation, customization, and dynamic responsiveness across its infrastructure, execution, and workload management. These include:
  Programmable: Measured by the feasibility of creating observatories using infrastructure as code, policy as code, and formal language.
  Configurable: Measured by I/O, deployment, code execution, and non-functional requirements.
  Adaptability: Assessed for infrastructure, I/O, code, and workload.
2. *Traditional Properties*: Measure the feasibility of creating static FAIR repositories and observatories.
3. *Value Properties for Resiliency*: Measure the system’s reliability, transparency, and collaborative capability, ensuring secure operations, fault tolerance, reproducibility, dynamic visualization, and safe sharing of resources. These include:
  Security: Evaluated by confidentiality, integrity, and access control.
  Fault-tolerance: Measured for data, metadata, and systems.
  Reproducibility: Measure the ability to recreate the same results or outcomes using the same methods, data, and conditions.
  Visualization: Assessed for on-the-fly/pre-calculated and responsive data visualization.
  Secure Sharing: Involving code, data, products, and services.

Recent initiatives such as ASCAPE^23^ and GEN-RWD Sandbox^24^ highlight the need for adaptable data platforms in healthcare and align with FAIR principles. However, they lack the modular programmability and PCA-level (Dynamic properties) and R-level (Value properties for resiliency) automation that *Jub* provides. Traditional development and deployment of a single FAIR repository or observatory on cloud services involves multiple actors such as architects, designers, engineers, and developers. Once deployed, analysts define information usability and build information products, while decision-makers consume the prepared products.

*Jub*, by contrast, introduces digital actors (e.g., *Jub Nodes*, *Jub Cloud*, *Jub Lake*), reducing participant intervention to analysts, scientific users, and decision-makers. This study observed a surge in *LHSDP* across various countries, with organizations implementing big data processing, visualization systems, and data science observatories. Table 1 compares PCA-FAIR-R feature compliance across notable examples from the literature and *Jub*. Rather than offering a static data platform, *Jub* proposes a constantly evolving platform guided by feedback from analysts, researchers, and decision-makers from *PCRN* and other healthcare and environmental organizations collaborating with the *Jub* project.

**Table 1:**
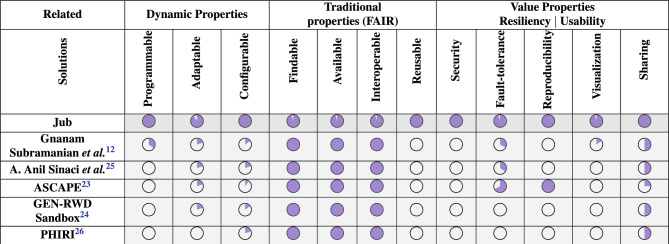
Life science and healthcare data platforms comparative table

Unlike existing solutions that are often monolithic or limited to static FAIR repositories, *Jub* introduces a modular and programmable approach through the use of sandboxes and configurable observatories. This architectural model allows dynamic fusion and profiling of heterogeneous datasets –such as spatial, temporal, clinical, and environmental data– into indexed, discoverable, and reusable information products. The open-source nature of *Jub* further supports community-driven development and reproducibility, aligning with FAIR and PCA-FAIR-R design goals.

## Conceptual architecture

*Jub* aims to facilitate data sharing across various public organizations, even in different geographical locations, to create a global hub of information products from diverse data sources (e.g., cancer mortality, environmental issues, climate). *Jub* aims to process extensive datasets to offer different views (e.g., maps, graphs, various data formats), enhancing resources for decision-makers to perform *DDDM* in strategic systems such as healthcare surveillance, earth observation, and territorial event monitoring.

**Fig. 1:**
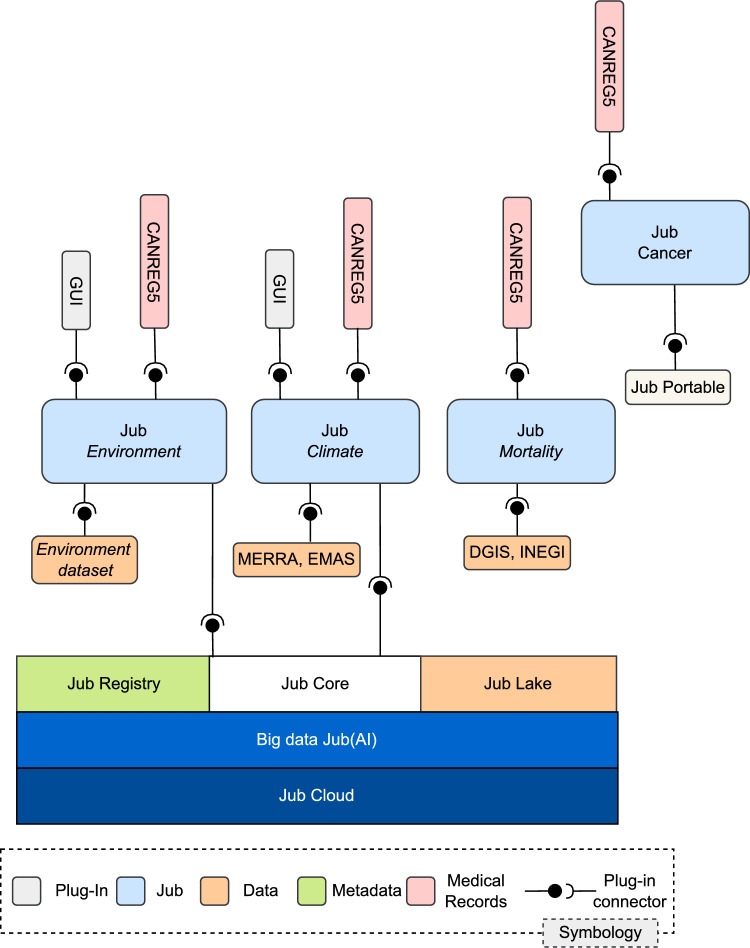
Overview of *Jub*’s architecture and data integration flow. The architecture is composed of core infrastructure services (*Jub Cloud*, *Big Data Jub (AI)*, *Jub Core*, *Jub Lake*, and *Jub Registry*) that support the creation and deployment of domain-specific observatories such as *Jub Environment*, *Jub Climate*, *Jub Mortality*, and *Jub Cancer*.

*Jub* relies on a generic cloud-based data model to build scalable, extensible, and adaptable data science systems manifested as information product observatories, which users can develop and configure. These observatories generate large sets of information products accessible through a retrieval system, allowing organizational users to discover insights. Figure 1 illustrates the conceptual architecture of *Jub* implemented with microservices.

The architecture is composed of modular services designed for scalability, reproducibility, and openness. All components are deployed in cloud-based environments collectively referred to as *Jub Cloud*, which provides the containerized infrastructure required for storage, computing, and orchestration. Within this environment, *Jub Core*–implemented in Python–coordinates the lifecycle of observatories, managing registration, profiling, and indexing workflows via a REST API. It serves as the integration point between user-defined sandboxes and the system’s data infrastructure. Data storage and indexing are handled by *Jub Lake*, a hybrid layer combining a Rust-based backend for high-throughput object storage with a Python-based client for interoperability and automation. All structured and unstructured data products are persistently stored and indexed for rapid retrieval. Complementing this, the *Jub Registry* acts as a centralized hub of reusable assets, including configuration templates, metadata schemas, and deployment blueprints for observatories. User interaction with the platform is facilitated through the *Jub GUI*, a frontend primarily developed in Vue.js. It provides access to dynamic visualizations, advanced querying, and responsive data exploration. The GUI is decoupled from the backend services, allowing its replacement or extension with alternative frameworks if needed. The platform also integrates with *Big Data Jub (AI)*, an external service that hosts configurable data mining and profiling algorithms. This service operates independently and can be swapped for custom analytics backends depending on the domain requirements. All these components are deployable both in the cloud and as a centralized, offline —*Jub Portable* edition—, ensuring compatibility with security-restricted environments. Specialized *Jub Nodes* (e.g., *Jub Mortality*, *Jub Environment*) represent domain-specific sandboxes built atop this infrastructure, enabling reproducible FAIR-compliant observatories with high configurability and domain adaptation.

These sandboxes support three types of connectors:

1. *Dataset Connectors*: Allow data extraction, integration, and fusion (orange connectors in Fig. 1).
2. *Interoperability Connectors*: Facilitate automatic dataset integration, sandbox tools, and user/organization applications (green connectors for interfaces, visualizations, panels, and web pages).
3. *Cloud Connectors*: Link sandboxes with the *Jub* platform through *Jub Core* (black connectors in Fig. 1), enabling the sharing of data, software, and pre-configured sandboxes for result reproduction.

This architecture underpins the implementation of *Jub*, simplifying the integration of data creation, indexing, storage, and visualization for massive data volumes. It also fosters collaborative research by deploying cohesive systems in generic sandboxes. The platform effectively delivers data science as a service, allowing organizations to select sandboxes and connectors for their assets. *Jub* empowers diverse stakeholders to transform raw data into meaningful insights for life sciences research and clinical applications. All core components of the platform–*Jub Core*, *Jub Cloud*, *Jub Lake*, observatory and repository builders, and GUI–are implemented as independently deployable open-sourced microservices.

***Jub Usage***

**Fig. 2:**
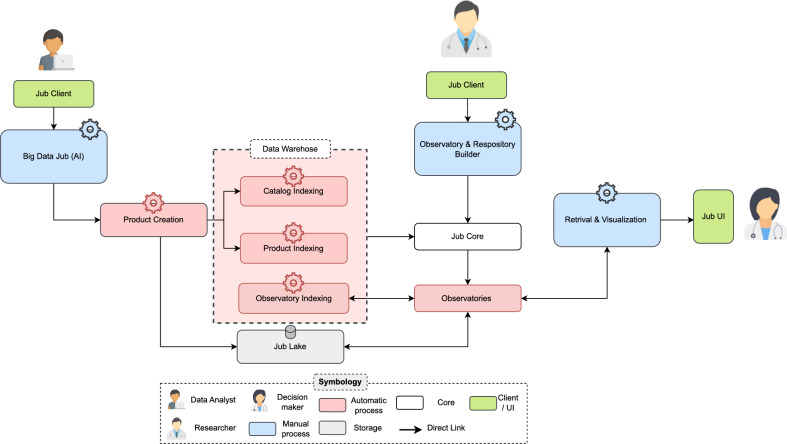
*Jub* platform architecture illustrating user interactions and roles. Data analysts generate information products, which are indexed and stored in *Jub Lake*. Researchers create observatories using the builder, and decision-makers access insights through the retrieval and visualization interface.

Figure 2 illustrates how *Jub sandboxes* are utilized within *Jub*. The user’s role is simplified into three primary actions: (1) Adding cured data sources, (2) Selecting the processing method and variables for study, and (3) Discovering information products through searches in online PCA-FAIR-R observatories, these PCA properties are implemented as operational extensions that work alongside FAIR guidelines. For example, programmability refers to the ability to instantiate observatories using infrastructure-as-code, while configurability allows analysts to tune datasets and interface behavior without modifying core system code.

*Jub* is a versatile and reusable *LSHDP* that automates these actions, ensuring transparent, efficient data sharing, storage, and processing at scale. In a typical scenario, users introduce cured data sources to *Jub*, which organizes and prepares the data. The platform then coordinates the data mining processes to extract insights and transform them into information products formatted for decision-makers to support their *DDDM*.

*Jub* supports three user types: Data Analysts, Researchers, and Decision-makers, each with tailored access to specific system components:

1. *Data analysts*: Utilize the *Big Data Jub (AI)* module via a *Jub Client* to create data products and conduct data analyses. These products are processed and organized within a Data Warehouse, comprising three indexing modules:
  *Catalog Indexing*: Organizes metadata.
  *Product Indexing*: Manages data products.
  *Observatory Indexing*: Indexes observatories as services.
  These indexed datasets are stored in *Jub Lake*, a centralized repository for scalable, flexible raw and processed data storage.
2. *Researchers*: Interact with *Jub* through the *Observatory and Repository Builders* to create observatories and repositories tailored to specific research domains. These observatories are linked to the *Jub Core*, the central coordination hub for managing and accessing observatory data. The *Jub Core* integrates observatory data and facilitates seamless retrieval for end-users.
3. *Decision-makers*: Access insights through the *Jub-GUI*, a user-friendly data retrieval and visualization interface. This interface ensures that insights are presented clearly and accessible, supporting *DDDM*.

*Jub* supports a wide variety of data modalities, including:

- **Structured data:** such as tabular records from registries.
- **Time-series data:** including environmental or physiological sensor signals.
- **Medical imaging:** such as histopathological images or CT scans (e.g., bone cancer datasets).
- **Unstructured or semi-structured data:** including ICD-10 classifications, textual records, and metadata schemas.

This multimodal capability allows for integrative analysis across domains such as epidemiology, pollution monitoring, and cancer research.

Visualizations in *Jub* are indexed through a REST API that allows querying indexed profiles by variables such as date, ICD-10 category, pollutant, and geography. Observatories expose endpoints for retrieving both raw data and precomputed information products, enabling seamless integration with frontends or external analysis tools. *Jub* simplifies data processing and retrieval by integrating these functionalities, empowering users to derive actionable insights from diverse data sources.

To ensure the platform remains domain-agnostic and scalable to new research questions beyond specific case studies, *Jub* implements a generic data model. This model relies on a hierarchical, faceted classification system that harmonizes heterogeneous data sources through three core mechanisms:

1. **Harmonization via Levels:** The platform uses controlled lists of terms (*Catalogs*), defined by the user, to organize data into a unified structure. Instead of relying on specific database schemas for every new domain, the system maps different datasets to generic **Levels**. For example, a standard configuration assigns Level 0 to *Time*, Level 1 to *Place*, and Level 2 to the *Topic*. This allows the core system to process many different data types without needing code changes.
2. **Configurable Observatories:** Users define an *Observatory* using a simple configuration file (JSON or YAML) rather than writing compiled code. This file dictates which lists of terms are valid for a specific research area. To add a completely new dataset, a user simply creates a new configuration file and defines the necessary catalogs. This design allows the platform to expand into new domains without modifying the *Jub Core* software.
3. **Flexible Data Processing:** The *Big Data Jub (AI)* module runs programmable pipelines that transform raw inputs–such as CSV files, sensor logs, or images–into easy configurable *Information Products*. These pipelines are modular, meaning researchers can easily swap them or write custom scripts to clean and prepare their specific data. This ensures that new data types are always compatible with the platform’s storage and retrieval systems.

This approach ensures that *Jub* effectively decouples data storage from semantic organization, allowing the platform to handle completely new datasets by simply updating configuration assets rather than re-engineering the system architecture.

## Case study: cancer mortality and air pollutants in Mexico

We conducted a case study involving three categories of *Jub* users who generated and utilized digital information products. The first group, *analysts*, defined methodologies for creating products like maps, graphs, and tables based on metrics such as per capita mortality rates. The second group, *researchers*, accessed information through the *Jub* retrieval system. This allowed them to identify patterns and trends, compiling reports that *decision-makers* can evaluate to support *DDDM*. This case study demonstrates *Jub*’s capacity to handle multimodal data, including large-scale structured mortality records, and geotemporal pollutant metrics. This validates the platform’s flexibility and generalizability across domains.

### Dataset description

This study focuses on researcher users who identify patterns and cross-reference data from two primary datasets forming two *Jub* observatories:

- ICD10-Mortality: This dataset is derived from the death registry of Mexico’s General Directorate of Health Information (DGIS, by its acronym in Spanish^27^). It includes temporal variables (2000–2022), spatial variables (states and municipalities), and interest variables (death types classified under the International Classification of Diseases - ICD-10^28^, sex, and age). We focused on per capita mortality ratios measured as deaths per 100,000 inhabitants at the state level and per 10,000 inhabitants at the municipal level.
- Air Pollutants: This dataset is sourced from Mexico City’s Automatic Atmospheric Monitoring Network (RAMA, by its acronym in Spanish^29^) and covers temporal variables (2004–2022) and spatial variables (municipalities). Interest variables include air pollutants (*CO*, *NO*, *PMCO*, $SO_2$, $PM_{2.5}$, $O_3$, $NO_x$, $PM_{10}$, and $NO_2$), with emissions measured in $\mu g/m^3$.

### Data visualization and analysis

Our strategy demonstrates *Jub* usage through a three-phase method for analysts:

1. *Analysts* use *Jub* to select observatory-relevant information products and generate variable combinations (temporal, spatial, and interest).
2. The platform computes observation variables for all combinations, producing corresponding information products.
3. Products are published to the *Jub Cloud*, registered in the *Jub Registry*, and stored in the *Jub Lake*.

**Fig. 3:**
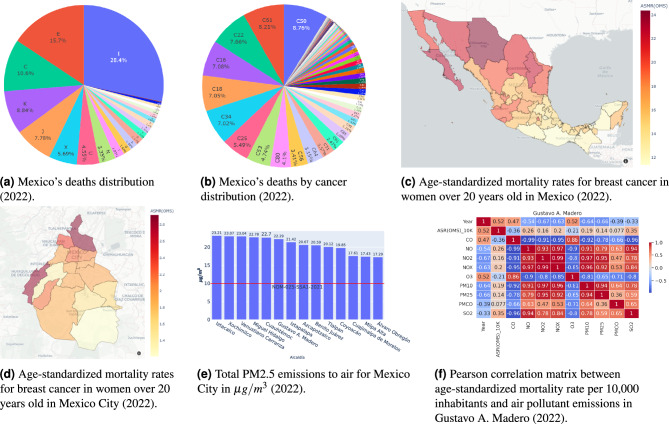
Representative visualizations from the *ICD10-Mortality* and *Air Pollutants* observatories. (**a**) General cause-of-death distribution in Mexico for 2022. (**b**) Distribution of cancer-related deaths by ICD-10 category. (**c**) National map of age-standardized breast cancer mortality rates in women over 20 years old. (**d**) Municipal-level breast cancer mortality rates in Mexico City. (**e**) PM2.5 emission levels across Mexico City municipalities, with a red line indicating the government threshold. (**f**) Pearson correlation matrix between air pollutants and breast cancer mortality rates in Gustavo A. Madero. All figures were produced by the authors employing the open-source data-visualization libraries Plotly v5.16.1 (https://plotly.com/python/) and Seaborn v0.13.2 (https://seaborn.pydata.org/.

Two observatories, *ICD10-Mortality*, and *Air Pollutants* were established within *Jub Core*, as shown in Fig. 3. *Researchers* employed *Jub-GUI* interfaces for data visualization, enabling data exploration by selecting variable values. For example, Fig. 3ashows the distribution of deaths in Mexico by general cause for 2022. The category “C” (cancer-related deaths) represented $10.6\%$ of total deaths.

Filtering by the code “C” allowed retrieval of relevant products from *Jub*. Figure 3billustrates the 2022 cancer mortality distribution by type, revealing breast (C50) and prostate cancer (C61) as the leading causes. *Researchers* further analyzed C50 mortality across Mexico (Fig. 3c), identifying high mortality rates in the northern border, *Guadalajara*, *Colima*, and *Mexico City (CDMX)*.

Fixing spatial variables to *CDMX* and analyzing municipalities revealed *Gustavo A. Madero*, *Azcapotzalco*, *Cuauhtémoc*, *Venustiano Carranza*, and *Cuajimalpa de Morelos* as having the highest breast cancer mortality rates (Fig. 3d). This analysis raises questions about potential links to lifestyle, healthcare disparities, or environmental factors. Identifying these influences is vital for public health *decision-makers* aiming to address mortality patterns.

To broaden the analysis, data fusion was employed by leveraging common temporal and spatial variables across datasets. For example, Fig. 3eshows $PM_{2.5}$ emissions for *CDMX* municipalities in 2022, exceeding the government’s $10 \mu g/m^3$ threshold^30^. Exploratory analysis, such as correlation matrices (Fig. 3f), revealed positive correlations between breast cancer mortality rates and pollutants like *NO*, $NO_2$, $PM_{10}$, $PM_{2.5}$, and $SO_2$ in *Gustavo A. Madero*, the municipality with the highest mortality rate in CDMX.

Further analysis can be conducted through *Jub*’s search and data visualization capabilities. Comprehensive research is necessary to establish definitive links between mortality rates and environmental factors, which falls beyond this study’s scope but is being explored in ongoing research.

This case study highlights the *Jub*’s capability to facilitate comprehensive data exploration and decision-making processes. By navigating across various profiles and observatory products, users can uncover trends, patterns, and relationships within complex datasets. This approach enhances data accessibility and empowers both expert and non-expert users to derive critical insights effectively.

**Fig. 4:**
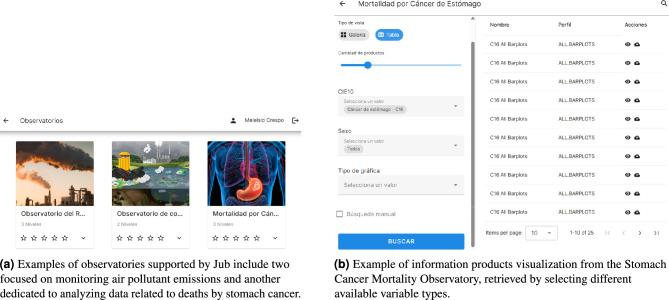
Examples of the graphical user interface designed and implemented for searching and displaying products within observatories. These illustrate how Jub dynamically generates a list of available observatories and enables users to explore the various product types within each one.

To support user accessibility and exploration, the platform includes a web-based interface (*Jub GUI*), primarily developed in Vue.js. The GUI (illustrated in Fig. 4) supports interactive exploration through domain-specific observatories. Users can navigate information products by selecting variable combinations via dropdowns or sliders, and visualize them through maps, time-series, correlation matrices, etc. The layout is modular and configurable, allowing field-specific interfaces for domains such as cancer, climate, or environmental health. Observatories are rendered using dynamic panels that can be extended or adapted through declarative configuration files, enabling reuse across research fields.

The novelty of the results presented in this case study lies in the integration of two heterogeneous, large-scale public datasets —structured mortality records from Mexico’s health authorities and air pollutant data from Mexico City’s atmospheric monitoring network— within a unified, interactive platform that enables user-driven exploration, analysis, and visualization. Unlike conventional epidemiological studies that focus on static, pre-defined relationships, this approach allows researchers and decision-makers to dynamically generate information products by combining temporal, spatial, and interest variables. This fine-grained integration facilitates, in this example, exploratory correlation analyses between environmental exposures and health outcomes, such as the observed associations between air pollution and breast cancer mortality in Mexico City. *Jub* supports accessibility through a modular, web-based graphical interface that requires no coding skills, enabling diverse users to interact with complex datasets intuitively. Moreover, the observatory model underpinning the system is declaratively defined and easily reusable, allowing rapid adaptation to other domains. By operationalizing open data through automated indexing, flexible visualizations, and domain-specific observatories, this work demonstrates a scalable and replicable framework for data-driven decision-making in public health and environmental research, particularly valuable in contexts like Latin America, where interoperable, user-centric data platforms remain scarce.

## Learned lessons

The key lessons learned from implementing observatories guided by the *PCRN* and other healthcare and environmental organizations were that observatories should integrate data science services with dynamic retrieval systems into a single reusable solution. These observatories need to address specific questions. By basing the observatories on profiling methodologies in *Jub*, they gained greater dynamism and had more influence from decision-makers while reducing the intervention of cloud provider staff.

To address these needs, *Jub*’s architecture was designed to be a dynamic, evolving big data science service. Collaboration with *PCRN* helped apply security measures to processes and data while adapting this proposal to create dynamic platforms. This approach avoided vendor lock-in issues commonly associated with static cloud-based solutions. From our experience exchanging feedback with decision-makers using these information products, we identified important lessons:

- Static information product availability is insufficient to conduct studies, as these products rely on specific variables. The *LSHDP* should present information products for multiple interest and observation variable combinations. Based on this experience, *Jub* was designed with a recursive computational model to calculate numerous combinations, producing vast information products to improve discovery.
- Indexing reference variables is essential for researchers when answering questions. *Jub*’s computational model is based on indexing reference variables to enhance searchability and retrieval.
- *DDDM* requires multiple visualizations of information products. *Jub* supports configurable dynamic panels to display multiple products simultaneously.
- Reusing data analysis methods is important. *Jub* allows users to select and execute existing methods to process datasets primarily used by analysts rather than decision-makers.

## Conclusions

This paper introduced *Jub*, a *LSHDP* leveraging generic analytic sandboxes that seamlessly integrate data analysis tools and cloud storage into data science services. These services empower organizations to conduct and customize complex analyses by automatically merging datasets to generate extensive information products across various scientific domains.

The case studies demonstrated *Jub*’s utility in uncovering spatial-temporal patterns and identifying pattern similarities across datasets, such as mortality and pollutants. These findings highlighted *Jub*’s effectiveness in helping organizations build multiple PCA-FAIR-R cloud-based observatories and process massive amounts of data to create a robust hub of information products. Moreover, *Jub* has proven valuable for supporting *DDDM* and enabling researchers to perform descriptive, prospective, and diagnostic studies efficiently.

## Data Availability

The raw datasets analyzed during the current study are available in the following public repositories: ICD-10 Mortality, http://www.dgis.salud.gob.mx/contenidos/basesdedatos/Datos_Abiertos_gobmx.html. Air Pollutants, https://datos.cdmx.gob.mx/dataset/red-automatica-de-monitoreo-atmosferico.

## A Observatories and information product inventory

Table 2 summarizes the distribution of information products across observatories and datasets. Key metrics include total products, total file sizes, average file sizes, median file sizes, and standard deviations in file sizes. These observatories span diverse domains such as pollutants, cancer research, mortality, and state-specific datasets, highlighting the reusability of *Jub* sandboxes and their configurable properties.

**Table 2:** Summary of information products generated across *Jub* observatories. The table illustrates the volume and variability of data outputs, highlighting the platform’s ability to manage diverse domains (e.g., cancer, mortality, environmental health) through configurable sandboxes and profiling workflows.

Observatory	Total Products	SUM. FILE_SIZE	AVG. FILE_SIZE	MEDIAN FILE_SIZE	STDV FILE_SIZE	Dataset
Pollutants	488	0.05 GB	0.09 MB	0.12 MB	0.07 MB	Pollutants
Benzene	450	0.29 GB	0.65 MB	0.09 MB	1.50 MB	Stomach Benzene
Stomach Cancer	16	0.03 GB	1.73 MB	1.73 MB	1.73 MB	Stomach Cancer
Pollutants	863	2.88 GB	3.42 MB	3.42 MB	0 MB	Pollutants
Tachometer	38468	0.3 GB	0.01 MB	0.01 MB	0 MB	Tachometer
RTC Puebla	38468	0.34 GB	5.28 MB	0.06 MB	7.68 MB	RTC Puebla
RTC Puebla	270	0.34 GB	0.01 MB	0.02 MB	0.01 MB	RTC Puebla
Mortality	30,229	77.36 GB	2.56 MB	1.08 MB	2.63 MB	Mortality
Elderly	14,886	49.32 GB	3.31 MB	3.60 MB	977.34 KB	Mortality
Cancer	2,682	6.92 GB	2.58 MB	604.86 KB	2.78 MB	Mortality
Bone Cancer	9,290	1.09 GB	116.90 KB	126.61 KB	41.75 KB	Bone Cancer Images
Contaminants	342	1.23 GB	3.59 MB	3.59 MB	1.81 KB	RAMA
CDMX	13,028	53.58 GB	4.11 MB	3.60 MB	1.78 MB	Mortality
Veracruz	1,918	36.32 GB	18.94 MB	3.60 MB	37.42 MB	Mortality
State of Mexico	24,550	120.12 GB	4.89 MB	3.60 MB	8.25 MB	Mortality
Chiapas	1,272	27.69 GB	23.65 MB	11.35 MB	23.07 MB	Mortality
**SUM**	**98,197**	**373.63 GB**	**63.75 MB**	**31.15 MB**	**76.96 MB**	
**AVG**	**10,910.78**	**41.51 GB**	**7.08 MB**	**3.46 MB**	**8.55 MB**	
**MEDIAN**	**9,290**	**36.32 GB**	**3.59 MB**	**3.60 MB**	**2.63 MB**	
**STDV**	**10,812.74**	**39.28 GB**	**8.25 MB**	**3.30 MB**	**13.05 MB**	

## Code availability

The source code of the Jub platform, including the Jub Core, Jub Lake, and Jub AI microservices, is available under the *BSD 3-Clause open-source license*. The live repositories are hosted at the dedicated GitHub Organization: https://github.com/jub-lshdp. To facilitate immediate reproducibility, pre-built Docker container images are available on Docker Hub under the namespace *nachocode/jub*, specifically: *nachocode/jub:api-0.0.1a0*, *nachocode/jub:gui-0.0.1a0*, *nachocode/jub:cloud-0.0.1a0*, *nachocode/jub:lake_router-0.0.1a0*, *nachocode/jub:lake_rm-0.0.1a0*, *nachocode/jub:lake_peer-0.0.1a0*, and *nachocode/jub:lake_summoner-0.0.1a0*. A comprehensive step-by-step guide for deploying the platform and reproducing the results presented in this manuscript is available in the *usage-guide* repository: https://github.com/jub-lshdp/usage-guide.

## References

[1] Luccio, D. D. et al. A high-performance, parallel, and hierarchically distributed model for coastal run-up events simulation and forecasting. *J. Supercomput.* **80**, 22748–22769. 10.1007/s11227-024-06188-5 (2024).

[2] Deka, B.et al. Rico: A mobile app dataset for building data-driven design applications. In *Proceedings of the 30th annual ACM symposium on user interface software and technology*, 845–854, 10.1145/3126594.3126651 (2017).

[3] Emilio, M. Di Paolo. Data acquisition systems. Fundamentals to Applied Design; *Springer: New York, NY, USA*, (2013).

[4] Bi, W. L. et al. Artificial intelligence in cancer imaging: Clinical challenges and applications. *Ca-Cancer J. Clin.* **69**, 127–157, 10.3322/caac.21552 (2019). 10.3322/caac.21552 PMC6403009 30720861

[5] for Research on Cancer (IARC), T. I. A. Global Cancer Observatory – gco.iarc.fr. https://gco.iarc.fr/. **Accessed 05/12/2024**.

[6] Caufield, J. et al. Kg-hub-building and exchanging biological knowledge graphs. *Bioinformatics*10.1093/bioinformatics/ (Oxford University Press (OUP), 2023) . 10.1093/bioinformatics/btad418 PMC10336030 37389415

[7] Oettl, F., Tischer, T., Wittmeir, T. & Schilp, J. From data to decisions: a method for evaluating the strategic value of digital twins. In *2023 3rd International Conference on Electrical, Computer, Communications and Mechatronics Engineering (ICECCME)*, 1–5, 10.1109/ICECCME57830.2023.10252781 (IEEE, 2023).

[8] Dong, X.L. et al. From data fusion to knowledge fusion. arXiv preprint arXiv:1503.00302(2015), 2015.

[9] Barron-Lugo, J. A., Gonzalez-Compean, J. L., Carretero, J., Lopez-Arevalo, I. & Montella, R. A novel transversal processing model to build environmental big data services in the cloud. *Environ. Model. Softw.* **144**, 10.1016/j.envsoft.2021.105173 (2021).

[10] Barron-Lugo, J. A., Gonzalez-Compean, J. L., Lopez-Arevalo, I., Carretero, J. & Martinez-Rodriguez, J. L. Xel: A cloud-agnostic data platform for the design-driven building of high-availability data science services. *Future Gener. Comput. Syst.* **145**, 87–103. 10.1016/j.future.2023.03.019 (2023).

[11] Tang, D. et al. Srplot: A free online platform for data visualization and graphing. *PLoS ONE* **18**, (2023).10.1371/journal.pone.0294236 10.1371/journal.pone.0294236 PMC10635526 37943830

[12] Subramanian, G. & Ramamoorthy, K. From data to diagnosis exploring aws cloud solutions in multi-omics breast cancer biomarker research. *Comput. Biol. Bioinform.* **12**, 1–11. 10.11648/j.cbb.20241201.11 (2024).

[13] Jarquin-Yañez, L., Cruz, E. T., Martinez-Acuña, M. I. & Calderon-Hernandez, J. Perceptions and attitudes about the contribution of the environment to childhood cancer: a pilot study in a medical guild and undergraduate students. *BMC Med. Educ.* **24**, 1138. 10.1186/s12909-024-05914-0 (2024). 39402539 10.1186/s12909-024-05914-0 PMC11476317

[14] González-García, J. et al. Phiri: Lessons for an extensive reuse of sensitive data in federated health research. *Eur. J. Public Health* **34**, i43–i49. 10.1093/eurpub/ckae036 (2024). 38946447 10.1093/eurpub/ckae036 PMC11215320

[15] Rujano, M. A. et al. *Sharing sensitive data in life sciences: an overview of centralized and federated approaches*(2024). 10.1093/bib/bbae262 10.1093/bib/bbae262 PMC11151787 38836701

[16] Subramanian, G. & Ramamoorthy, K. From data to diagnosis exploring aws cloud solutions in multi-omics breast cancer biomarker research. *Comput. Biol. Bioinform.* **12**, 1–11. 10.11648/j.cbb.20241201.11 (2024).

[17] Krishnasamy, S. My holistic data share: A web3 data share application: Extending beyond finance to privacy-protected decentralized share of multi-dimensional data to enhance global healthcare. *Blockchain Healthc. Today* **7**, (2024).10.30953/bhty.v7.341 10.30953/bhty.v7.341 PMC11624492 39649411

[18] Opara-Martins, J., Sahandi, R. & Tian, F. Critical analysis of vendor lock-in and its impact on cloud computing migration: a business perspective. *J. Cloud Comput.* **5**, 1–18 (2016).

[19] Hong, N. P. C. et al. Fair principles for research software (fair4rs principles). *Zenodo* (2022).

[20] Lamprecht, A.-L. et al. Towards fair principles for research software. *Data Sci.* **3**, 37–59 (2020). 10.1038/s41597-022-01710-x PMC9562067 36241754

[21] Singh, P. *Learn PySpark: Build Python-based Machine Learning and Deep Learning Models* (Apress, 2019), 1 edn.

[22] Barron-Lugo, J. A., Gonzalez-Compean, J., Lopez-Arevalo, I., Carretero, J. & Martinez-Rodriguez, J. L. Xel: A cloud-agnostic data platform for the design-driven building of high-availability data science services. *Future Gener. Comput. Syst.* **145**, 87–103 (2023).

[23] Ivanovic, M., Autexier, S., Kokkonidis, M. & Rust, J. Quality medical data management within an open ai architecture – cancer patients case. *Connect. Sci.* **35**, (2023). 10.1080/09540091.2023.2194581

[24] Gottardelli, B. et al. Gen-rwd sandbox: bridging the gap between hospital data privacy and external research insights with distributed analytics. *BMC Med. Inform. Decis. Mak.* **24**, 170. 10.21203/rs.3.rs-3816282/v1 (2024). 38886772 10.1186/s12911-024-02549-5 PMC11184891

[25] Sinaci, A. A. et al. A data transformation methodology to create findable, accessible, interoperable, and reusable health data: Software design, development, and evaluation study. *J. Med. Internet Res.* **25**, e42822. 10.2196/42822 (2023). 36884270 10.2196/42822 PMC10034606

[26] González-García, J. et al. Phiri: lessons for an extensive reuse of sensitive data in federated health research. *Eur. J. Public Health* **34**, i43–i49. 10.1093/eurpub/ckae036 (2024). 38946447 10.1093/eurpub/ckae036 PMC11215320

[27] Dirección General de Informacióón en Salud. Defunciones datos abiertos. http://www.dgis.salud.gob.mx/contenidos/basesdedatos/Datos_Abiertos_gobmx.html. **Accessed on 02/13/2024**.

[28] World Health Organization. International statistical classification of diseases and related health problems. https://www.who.int/standards/classifications/classification-of-diseases. **Accessed: 01/20/2025**.

[29] Secretaríía del Medio Ambiente. Red automáática de monitoreo atmosférico (rama). https://datos.cdmx.gob.mx/dataset/red-automatica-de-monitoreo-atmosferico. **Accessed: 01/22/2025**.

[30] Secretaría de Salud. Norma oficial mexicana nom-025-ssa1-2021. https://www.dof.gob.mx/nota_detalle.php?codigo=5633855&fecha=27/10/2021##gsc.tab=0 **Accessed: 01/22/2025**.

